# Glycaemic screening before kidney transplantation should include an oral glucose tolerance test

**DOI:** 10.3389/fneph.2026.1849545

**Published:** 2026-10-23

**Authors:** Espen Nordheim, Trond Geir Jenssen, Jørn Petter Lindahl, Kåre Inge Birkeland, Karsten Midtvedt, Kristian Heldal, Morten Hagness, Markus Hovd, Anders Åsberg

**Affiliations:** 1 Department of Transplantation Medicine, Oslo University Hospital, Rikshospitalet, Oslo, Norway; 2 Institute of Clinical Medicine, University of Oslo, Oslo, Norway; 3 The Norwegian Renal Registry, Oslo University Hospital, Rikshospitalet, Oslo, Norway; 4 Department of Pharmacy, University of Oslo, Oslo, Norway

**Keywords:** Diabetes Mellitus, Type 2 diabetes, Kidney transplantation, Screening, Alternative 1 "oral glucose tolerance test", Alternative 2 "glucose", Alternative 3 "transplantation"

## Introduction

The presence of diabetes mellitus (diabetes) adversely affects outcomes after kidney transplantation, and glycemic screening should be included in the pretransplant evaluation of kidney transplant recipients. However, there is limited evidence regarding the optimal glycemic screening protocol, and current practices vary. According to the diagnostic criteria defined by the American Diabetes Association, diabetes may be diagnosed based on HbA1c or plasma glucose criteria, including either fasting plasma glucose or 2-h plasma glucose during a 75-g oral glucose tolerance test (1) (OGTT). In asymptomatic patients who fulfil the diagnostic criteria for diabetes, confirmatory testing is needed to establish the diagnosis (1). The KDIGO guidelines regard OGTT as the gold standard for pretransplant assessment of glycemia (2). However, a European survey from the ERA DESCARTES Working Group indicates that this recommendation has not been universally implemented (3). As part of the pretransplant workup in Norway, all kidney transplant candidates without known diabetes routinely undergo assessment with both HbA1c and OGTT.

This study aimed to determine the frequency and distribution of diabetes-range HbA1c, fasting plasma glucose, and 2-h plasma glucose results among kidney transplant recipients without known diabetes. The assessments were made according to existing pretransplant protocol at the time the patients were evaluated for waitlisting.

## Methods

In this retrospective observational study, patients who received a kidney transplant in Norway between 2007 and 2022 (*n* = 3,290) were identified from the national transplant registry. We excluded patients with preexisting diabetes (*n* = 724), patients listed for re-transplantation (*n* = 26) or combined organ transplantation (*n* = 22) as well as patients without available HbA1c or OGTT (*n* = 466), leaving 2,052 patients eligible for the study. Data describing HbA1c and OGTT were retrieved from the patients’ electronic medical records. All patients have provided signed informed consent covering this kind of analyses, and the study was approved by the Regional Committee for Medical and Health Research Ethics South-East (reference 407293). During the study period 2007–2022, 5.5% of patients died while waitlisted for kidney transplantation, and 11.9% were permanently withdrawn from the waiting list for kidney transplantation for various reasons. Among all patients waitlisted for kidney transplantation during the study period, 12 patients (0.4%) are still waiting for kidney transplantation.

The American Diabetes Association criteria for diabetes were used to define diabetes-range glycemic levels: HbA1c ≥48 mmol/mol or plasma glucose ≥7.0 mmol/L or 2-h plasma glucose ≥11.1 mmol/L (1).

The proportion of patients fulfilling the diabetes-range glycemic levels were reported as rates or percentages, and the distribution is illustrated in **Figure 1** using the combination of a Venn diagram and a scatter plot. Overlapping circles in the Venn diagram illustrate the rate of patients fulfilling more than one diagnostic criterion.

**Figure 1:**
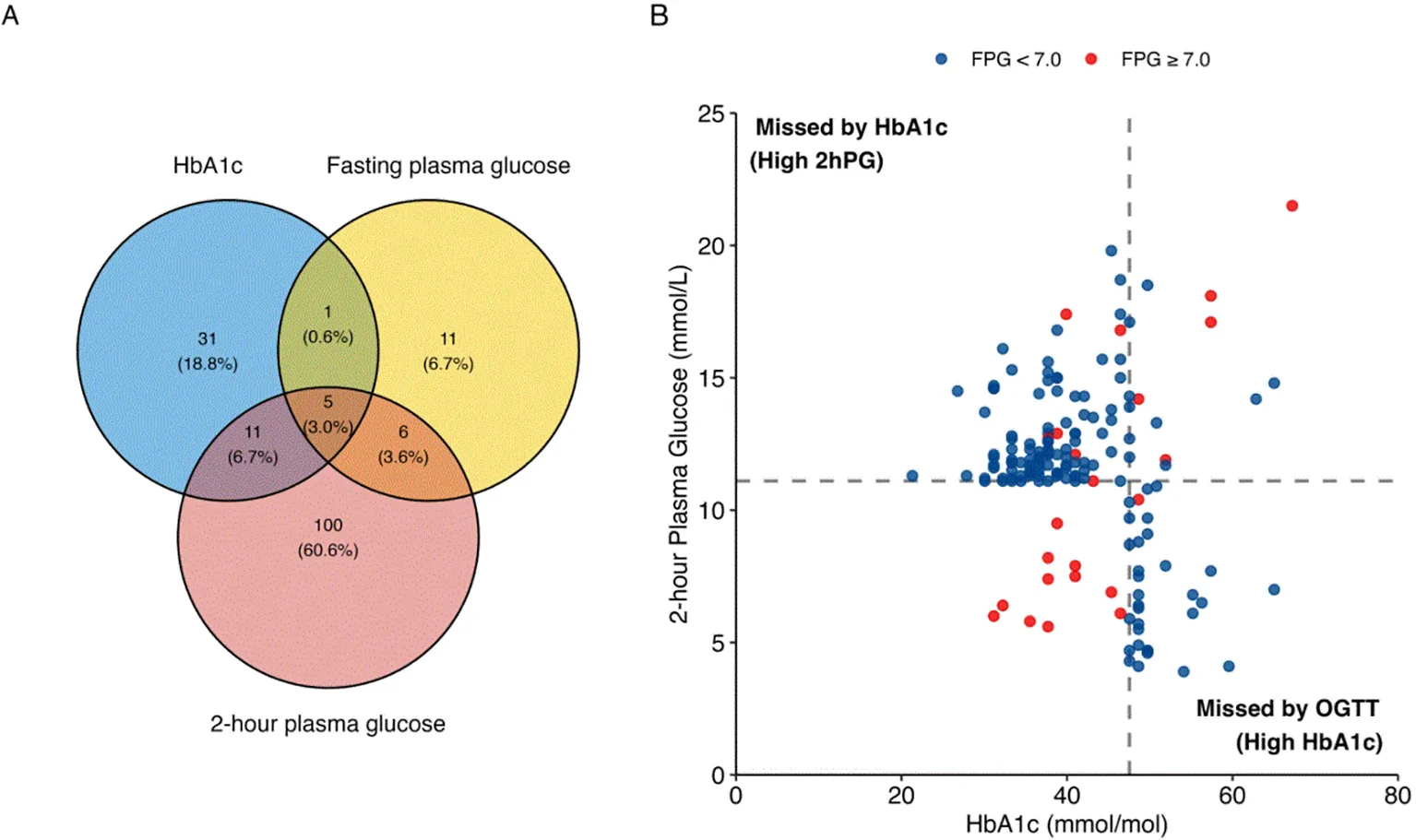
Distribution of 165 kidney transplant recipients without known diabetes patients fulfilling one or more criteria for diabetes-range glycemic levels in our study cohort illustrated in a Venn diagram **(A)** and scatter plot **(B)**, demonstrating concordance between the HbA1C and plasma glucose criteria in our study population.

The American Diabetes Association criteria for prediabetes were used to define prediabetes range glycemic levels (1): HbA1c from 39 to 47 mmol/mol or plasma glucose from 5.6 to 6.9 mmol/L or 2-h plasma glucose from 7.8 to 11.0 mmol/L.

As part of the pretransplant workup, HbA1c test and OGTT investigations were generally scheduled on a dialysis-free day by the local nephrologist. OGTT test routines have been standardized in Norwegian hospitals throughout the study period. The OGTT was performed in the morning after at least 10 h of fasting with an oral administration of 75 grams of glucose in 200 mL of water when the patients were seated and had avoided smoking on the same day and were in their common medical state. They were instructed to stay on a weight-maintaining diet for 3 days prior to testing and abstain from alcohol. Plasma glucose was measured just prior to and 30 and 120 min after the glucose load, and HbA1c was also assessed in a pre-OGTT sample. HbA1c analyses have been standardized in Norwegian hospitals since 2007 with standardized high-performance liquid chromatography (HPLC) or immunochemical methods certified by the IFCC in accredited laboratories. The HbA1c and OGTT test results were sent to the national transplant center as part of transplant candidate evaluation. The year of glycemic testing, but not the exact date, was available for this study. In case of repeated tests, only data on the closest OGTT preformed prior to being waitlisted for transplantation were available in this analysis. The study represents a complete national cohort of eligible transplanted recipients during the study period.

## Results

This study included results from 2,052 kidney transplant recipients (1,354 men, 66%), with mean age at transplantation being 54 years (range: 18–83 years), and the mean BMI was 25.8 kg/m^2^ (range: 16.0–36.2 kg/m^2^). A total of 30% of the patients were transplanted pre-emptively, while 47% were in hemodialysis and 23% were in peritoneal dialysis before transplantation. The mean time from the OGTT/HbA1c assessment to transplantation was 1.62 years (range 0–8).

We identified 165 kidney transplant recipients (8%) without known diabetes with glycemic test results within the diabetes range detected during pretransplant workup. Out of these, 48 patients fulfilled the HbA1c criterion and 23 patients fulfilled the fasting plasma glucose criterion, while 122 fulfilled the 2-h plasma glucose criterion (**Figure 1**). In 23 cases, more than one diagnostic criterion was met. Baseline BMI was comparable between included and excluded patients, but the excluded patients were, on average, 5 years younger than those included. Additional data are available in the **Supplementary Material**.

If the 2-h glucose tests results were excluded, only 65 patients (39%) were found to have glycemic test results within the diabetes range. This approach with HbA1c and/or fasting plasma glucose assessment only revealed 39% of the cases compared to when 2-h plasma glucose data were included in the analyses.

Among the remaining 1,887 patients without diabetes-range glycemic test results, 976 patients (52%) had test results within the range of prediabetes according to one or several ADA criteria (HbA1c criterion: *n* = 514, fasting glucose criterion: *n* = 417, and 2-h plasma glucose criterion: *n* = 449).

## Discussion

The main finding of our study was that 8% of Norwegian kidney transplant recipients without known diabetes were identified at the time for waitlisting as having pretransplant diabetes-range glycemic levels. These findings are in accordance with the results from two smaller studies from Austria (*n* = 138) and Spain (*n* = 186) which detected 3% and 6%, respectively, of patients with diabetes-range screening results among those waitlisted for kidney transplantation (4, 5). In our study, the vast majority (74%) were identified through the 2-h plasma glucose criterion. Most of these patients would likely have been missed for further diagnostics and follow-up if the pre-waitlisting OGTT screening had not been included. While some transplant centers prefer fasting glucose and HbA1c and not OGTT in this clinical setting, our findings support the inclusion of OGTT as the gold standard for pretransplant glucose assessment as recommended in the KDIGO guidelines (2). Discrepancies between HbA1c and OGTT test results have also been described in the general population (6) and among kidney-transplanted patients (7). Additionally, it is known that HbA1c has its limitations to correctly reflect glycemia in patients with chronic kidney disease (8). In the present study, we lack information on whether the pre-waitlisting test findings triggered a follow-up re-test and/or treatment of diabetes or preventive efforts in prediabetes, and we suggest that such measures should be implemented. Hyperglycemia identified before transplantation can be used to discuss a tailored approach to lifestyle or pharmacological interventions, when appropriate. In cases of overweight/obesity, weight loss can prevent diabetes to develop but also put diabetes into remission when identified early (9). Since both prediabetes and diabetes are associated with increased mortality and cardiovascular risk after kidney transplantation (10, 11), the pretransplant OGTT test results can be utilized in a continuous risk assessment for post-transplant diabetes among other post-transplant outcomes, as recommended by the 2022 PTDM Consensus Meeting (12). Some transplant centers consider the use of a less diabetogenic immunosuppressive regimen for patients at increased risk after kidney transplantation (13).

A strength of our study is the relatively large national cohort of Norwegian kidney transplant recipients without known diabetes, with available HbA1c and OGTT sampled as part of systemic and standardized pretransplant workup, which allowed this study to analyze the overlap among individual screening thresholds. Our study has some limitations. Treatment data on anemia, blood transfusions, erythropoiesis-stimulating agents, and iron supplementation were unfortunately not available for this study. Reported measurements of HbA1c and OGTT were performed only once per patient, while confirmative measurement is advocated in asymptomatic subjects before establishing a diabetes diagnosis (1). OGTT is well known to have limited reproducibility, and our study cohort included primarily data from patients who were transplanted and were predominantly Caucasian, limiting generalizability to all transplant candidates and other ethnic groups.

## Conclusion

In this cohort of kidney transplant recipients without previously known diabetes who underwent pretransplant glycemic assessment, 8% had at least one screening result within the diabetes range. Notably, most cases were identified through the 2-h plasma glucose criterion. These findings suggest that reliance on HbA1c and fasting plasma glucose alone may miss patients who warrant further diagnostics and follow-up for diabetes.
