# Supplementary material for: Glycaemic screening before kidney transplantation should include an oral glucose tolerance test
Source: Front Nephrol. 2026 Oct 23;6:1849545. doi: 10.3389/fneph.2026.1849545 (PMC13746163; doi:10.3389/fneph.2026.1849545)
Supplement: Supplementary file 1 [file DataSheet1.docx]

**Supplementary material on time from pretransplant OGTT/HbA1c assessment to kidney transplantation:**

Mean 1.62 years, median 1 year, range 0-8 years, standard deviation 1,3.
Quartiles listed in table below:

| Percentiles | |
| --- | --- |
| 25 | 1 |
| 50 | 1 |
| 75 | 2 |

397 candidates had glycaemic assessment performed more than 2 years before transplantation. 31 out of these had diabetes range glycaemic levels; 9 by HbA1c criterion, 4 by fasting glucose criterion and 24 by 2-hour criterion.

1655 candidates had glycaemic assessment performed within 2 years before transplantation. 134 out of these had diabetes range glycaemic levels; 39 by HbA1c criterion, 19 by fasting glucose criterion and 98 by 2-hour criterion.

1138 candidates had glycaemic assessment performed within 1 years before transplantation. 83 out of these had diabetes range glycaemic levels; 21 by HbA1c criterion, 10 by fasting glucose criterion and 65 by 2-hour criterion.

1430 patients received dialysis before kidney transplantation. Among these, time from glycaemic assessment till kidney transplantation was mean 1.81 years (SD 1.3), median 2 years and range 0-8 years.
133 out of these had diabetes range glycaemic levels; 39 by HbA1c criterion, 19 by fasting glucose criterion and 96 by 2-hour criterion.

622 patients were transplanted pre-emptively. Among these, time from glycaemic assessment till kidney transplantation was mean 1.12 years (SD 0.9), median 1 years and range 0-6 years.
32 out of these had diabetes range glycaemic levels; 9 by HbA1c criterion, 4 by fasting glucose criterion and 26 by 2-hour criterion.
The rate of patients with diabetes range glycaemic levels was higher among patients on dialysis before transplantation compared to patients transplanted pre-emptively; 9.3% vs 5.1 % (p<0.001).

Supplementary material on Distribution of 165 kidney transplant recipients without known diabetes patients fulfilling one or more criteria for diabetes- range glycaemic levels in our study cohort:
122 patients fulfilled 2-hour criterion; 6 of these also fulfilled the fasting glucose criterion, 11 fulfilled the HbA1c criterion while 5 patients fulfilled all three criteria.
